# Intraepithelial CD8-positive T lymphocytes predict survival for patients with serous stage III ovarian carcinomas: relevance of clonal selection of T lymphocytes

**DOI:** 10.1038/sj.bjc.6605274

**Published:** 2009-09-29

**Authors:** M Stumpf, A Hasenburg, M-O Riener, U Jütting, C Wang, Y Shen, M Orlowska-Volk, P Fisch, Z Wang, G Gitsch, M Werner, S Lassmann

**Affiliations:** 1 Department of Obstetrics and Gynecology, Albert-Ludwigs-University, Freiburg, Germany; 2 Institute of Pathology, University Medical Center, Albert-Ludwigs-University, Freiburg, Germany; 3 Institute of Clinical Pathology, University Hospital, Zurich, Switzerland; 4 Institute for Biomathematics and Biometry, HelmholtzZentrum München, German Center for Environmental Health, Neuherberg, Germany; 5 Department of Obstetrics and Gynecology, Tongji Medical College, Huazhong University of Science and Technology, Wuhan, China

**Keywords:** ovarian carcinoma, lymphocytes, survival, clonality

## Abstract

**Background:**

The aim of this study was to investigate the prognostic effect of tumour-infiltrating lymphocytes (TILs) in serous stage III ovarian carcinoma to determine TIL clonality and to correlate this to Her2/neu expression.

**Methods:**

Formalin-fixed and paraffin-embedded ovarian carcinomas were examined for CD20-, CD3-, CD4- and CD8-positive lymphocytes (*n*=100), and for Her2/neu-positive tumour cells (*n*=55/100) by immunohistochemistry. Clonality analysis was carried out by T-cell receptor *γ* (TCR*γ*) gene rearrangements (*n*=93/100). Statistical analyses included experimental and clinico-pathological variables, as well as disease-free (DFS) and overall (OS) survival.

**Results:**

CD20-positive B lymphocytes were present in 57.7% (stromal)/33.0% (intraepithelial) and CD3-positive T lymphocytes in 99.0% (stromal)/90.2% (intraepithelial) of ovarian carcinomas. Intraepithelial CD3-positive T lymphocytes were correlated with improved DFS in optimally debulked patients (*P*=0.0402). Intraepithelial CD8-positive T lymphocytes were correlated with improved OS in all optimally debulked patients (*P*=0.0201) and in those undergoing paclitaxel/carboplatin therapy (*P*=0.0092). Finally, rarified and clonal TCR*γ* gene rearrangements were detected in 37 out of 93 (39.8%) and 15 out of 93 (16.1%) cases, respectively. This was marginally associated with improved DFS (*P*=0.0873). Despite a significant correlation of HER2/neu status and intraepithelial CD8-positive lymphocytes (*P*=0.0264), this was non-directional (*R*=−0.257; *P*=0.0626).

**Conclusion:**

Improved survival of ovarian cancer patients is related to the infiltration, clonal selection and intraepithelial persistence of T lymphocytes.

Ovarian cancer is the leading cause of death from gynaecological cancers, and the fifth leading cause of cancer death in women (Runnebaum and Stickeler, 2001; Jemal *et al*, 2005). The majority (90%) of primary ovarian tumours derives from epithelial cells, the remainder arise from other cell types (e.g., germ cell tumours, sex cord-stromal tumours and mixed cell tumours) (Scott and McCluggage, 2006). As symptoms of early-stage disease are vague, and as valid markers for early-stage ovarian cancer are still missing, most patients are diagnosed in advanced stages (International Federation of Gynecology and Obstetrics (FIGO) stages III and IV) (Bhoola and Hoskins, 2006). The associated 5-year survival rate for advanced ovarian cancer is only 30–40% (Runnebaum and Stickeler, 2001; Pfisterer *et al*, 2005). Standard treatment involves surgical resection of the tumour with the goal of complete tumour reduction (optimal debulking) (Pfisterer *et al*, 2005) and adjuvant chemotherapy, with a standard combination of a taxane (e.g., paclitaxel and docetaxel) and a platinum (e.g., carboplatin and cisplatin) compound (du Bois *et al*, 2005; Bhoola and Hoskins, 2006). Established prognostic factors are stage, histologic subtype, tumour grade, the debulking status of the primary surgical resection and response to chemotherapy, which is measured in terms of disease-free survival (DFS) (Jemal *et al*, 2005; Bhoola and Hoskins, 2006). Reliable outcome prediction for DFS and overall survival (OS), however, still remains difficult, even for patients with similar clinico-pathological characteristics, and the search for valid clinical and/or molecular markers for prognosis and prediction of therapeutic response is ongoing.

Infiltration by immune cells (tumour-infiltrating lymphocytes (TILs)) is a central mechanism of the ‘hosts’ response to several types of human carcinomas and the TIL-mediated activity may be directed against tumour cell antigens (Parmiani, 2005). TILs have been recognised in several major human cancers, such as in adenocarcinomas of the lung (Dieu-Nosjean *et al*, 2008), the colorectum (Galon *et al*, 2006) and the breast (Sheu *et al*, 2008).

In the case of ovarian cancer (Zhang *et al*, 2003; Curiel *et al*, 2004; Raspollini *et al*, 2005; Sato *et al*, 2005; Hamanishi *et al*, 2007; Tomsová *et al*, 2008; Clarke *et al*, 2009; Leffers *et al*, 2009), the presence of T-lymphocyte infiltration in ovarian carcinomas has a major impact on the progression and clinical follow-up of patients. In particular, it has been shown that high numbers of CD3-positive T cells are indicative of improved survival (Zhang *et al*, 2003; Raspollini *et al*, 2005; Tomsová *et al*, 2008) and that the CD8-positive (cytotoxic) subtype of CD3-positive T lymphocytes is responsible for this effect (Sato *et al*, 2005; Hamanishi *et al*, 2007; Clarke *et al*, 2009; Leffers *et al*, 2009). In contrast, the presence of the CD4-positive regulatory subtype of T lymphocytes (Treg; CD4+CD25+FoxP3+) seems to reduce tumour-specific immunity and results in a poorer survival of patients with ovarian carcinomas (Curiel *et al*, 2004). Moreover, the exact location of TILs within the tumour mass has been shown to be important for the prognostic effect in ovarian cancer (Zhang *et al*, 2003; Sato *et al*, 2005; Clarke *et al*, 2009). As observed for colorectal cancers (Galon *et al*, 2006), ‘intratumoural’ infiltration by TILs is considered to be essential for revealing a prognostic impact.

Despite this wealth of information on TIL in ovarian carcinomas, several issues remain unclear. In particular, this applies to the definition and clinical impact of both ‘intra’- and ‘extra’-tumoural TILs for ovarian carcinoma patients treated with different adjuvant chemotherapy protocols. Moreover, the mechanism of TIL function within the tumour parenchyma requires further investigation. It may well be that ‘intratumoural’ TILs require recognition of tumour-specific antigens to become (re-)activated and persistent within the tumour parenchyma, as only then will they gain the ability to stimulate an anti-tumoural response. Clearly, if single tumour-cell-specific antigens are responsible for triggering this activation of TILs and the subsequent anti-tumoural responses, the activated TILs may be clonally restricted. This scenario has been proposed and experimentally supported in the case of HER2/neu-expressing tumour cells in breast carcinomas (Peoples *et al*, 1995; Wiech *et al*, 2008, Goodell *et al*, 2008). Evidence of such a mechanism for TILs in ovarian carcinoma is still sparse (Raspollini *et al*, 2005; Yang *et al*, 2007), especially as the rate of HER2/neu-expressing tumour cells varies from 1.9 to 35% (Press *et al*, 2005). Nevertheless, the identification of clonally restricted TILs represents an important basis for further exploitation and development of immune-based therapies (Sabbatini and Odunsi, 2007), also targeting Her2/neu (Disis *et al*, 2002). Although the identification of the target antigen (or antigens) recognised by prognostically beneficial TILs is highly complex, the specifically (re-)activated and hence clonal TILs can be examined by the determination of T-cell receptor (TCR) gene rearrangements. Similar approaches are used routinely for the molecularpathological analysis of suspected malignant lymphomas (van Dongen *et al*, 2003).

The aim of this study was to characterise the presence and exact localisation of TILs by immunohistochemistry in a homogeneous group of 100 serous FIGO stage III ovarian carcinoma patients treated by different adjuvant chemotherapy protocols. This allowed us to further examine and validate the prognostic value of stromal and intraepithelial TILs in clinically relevant sub-groups of ovarian carcinoma patients. Moreover, we analysed TCR chain rearrangements and Her2/neu expression to determine whether the generally accepted prognostic benefit of T lymphocytes in ovarian carcinomas (Zhang *et al*, 2003; Curiel *et al*, 2004; Raspollini *et al*, 2005; Sato *et al*, 2005; Hamanishi *et al*, 2007; Tomsová *et al*, 2008; Clarke *et al*, 2009; Leffers *et al*, 2009) is caused by a specific (re-)activation of individual clonally restricted TILs within the tumour parenchyma by HER2/neu-expressing tumour cells.

## Materials and methods

### Patients and tissues

The study included tissue specimens of 100 Caucasian patients with FIGO stage III serous ovarian carcinomas, treated between 1998 and 2005 at the Department of Obstetrics and Gynecology, University Hospital of Freiburg. The median clinical follow-up of patients was 22.5 months (range 1–82 months) and the major clinico-pathological data are given in Table 1. The terms ‘disease-free survival’ and ‘overall survival’ denote the time from the primary resection of ovarian carcinoma until the events of tumour recurrence and death due to ovarian cancer, respectively.

Formalin-fixed and paraffin-embedded tissue specimens of all cases were derived from routinely processed primary resection specimens (archive, Institute of Pathology, University Medical Center, Freiburg, Germany). Only tissue specimens of the central areas of ovarian carcinomas, that is, excluding metastases, were used. In addition, tissue specimens of 24 non-neoplastic, uninvolved ovaries from patients undergoing surgery for other purposes (for example, cysts) were included as normal controls. This study was approved by the local ethics committee (#97/05; Ethik-Kommission der Albert-Ludwigs-Universität, Freiburg, Germany). Routinely processed haematoxylin and eosin-stained tissue sections of invasive carcinomas were re-evaluated according to WHO (Tavassoli and Deville, 2003) and UICC (Sobin and Wittekind, 2002) classifications, and representative tissue areas of invasive carcinomas, reflecting about 60% tumour and 40% stroma content, were selected for the construction of tissue microarrays (TMAs) with core biopsies of 0.2 cm diameter.

### Immunohistochemistry

For immunohistochemical analysis, serial sections of TMAs were cut at 3-μm thickness, deparaffinised in xylene, graded alcohol (2 × 100; 95, 75, 50%) and water, and were subsequently subjected to antigen retrieval in Tris-EDTA-buffered or citrate-buffered solutions in a pressure cooker, according to routine protocols for the selected antibodies (CD20: pH 9.0, CD3: pH 9.0, CD4: pH 9.9, CD8: pH 6.0 and Her2/neu: pH 6.0). This was followed by routine staining on a DAKO Autostainer with primary antibodies (all from DAKOCytomation, with final dilution of CD20=1 : 200; CD3=1 : 200; CD4=1 : 500; CD8=1 : 200 and Her2/neu=1 : 350) for 60 min and by a secondary antibody incubation and detection using the LSAB-Fast red system (DakoCytomation, Glostrup, Denmark). Sections of formalin-fixed and paraffin-embedded normal tonsils and a breast cancer sample were run in parallel as positive controls.

In the ovarian carcinoma samples, scoring of CD20, CD3, CD4 and CD8 protein expression was carried out separately for intraepithelial and stromal (tumour-associated stroma and/or perivascular spaces) tumour areas (each three high-power fields (HPF) at × 40 magnification), with scores adapted from Zhang *et al* (2003): Score 0=no staining; score 1=fewer than five positive lymphocytes per HPF; score 2=5–20 positive lymphocytes per HPF and score 3=more than 20 positive lymphocytes per HPF. The evaluation of Her2/neu staining in invasive tumour cells was carried out according to routine diagnostic guidelines, with score 0=negative Her2/neu expression or incomplete membranous Her2/neu expression in <10% of invasive tumour cells; score 1=partial membranous Her2/neu expression in >10% of invasive tumour cells; score 2=weak, but complete membranous Her2/neu expression in >30% of invasive tumour cells and score 3=strong and complete membranous Her2/neu expression in >30% of invasive tumour cells.

In non-neoplastic ovaries, the same CD scoring system was applied for analysing the presence of lymphocytes (score 1–3) in three randomly chosen HPFs within the entire tissue section.

### Analysis of TCR *γ* gene rearrangements

For evaluation of T-cell clonality, each two 10-*μ*m serial sections of invasive ovarian cancers (*n*=100) and non-neoplastic ovaries (*n*=24) were subjected to deparaffination in xylene, graded alcohol (2 × 100; 95, 75, 50%) and water, followed by a brief staining in instant haematoxylin (Shandon, Thermo Scientific, Oberhausen, Germany). Using fine needles, invasive tumour cells (ovarian cancers) or ovarian stromal cells (non-neoplastic ovaries) were microdissected under a stereotactic microscope (Zeiss, Carl Zeiss, Göttingen, Germany) and subjected to DNA extraction according to protocols of the DNeasy Kit (Qiagen). DNA was eluted in a 100 *μ*l elution buffer and each 10 *μ*l DNA was used for PCR-based analysis of the TCR V*γ*1-8,10 (tube A) and V*γ*9,11 (tube B) chains using a modified protocol of Biomed-2 PCR and capillary electrophoresis analysis (van Dongen *et al*, 2003). In brief, for tube A 10 *μ*l of DNA was mixed with 25.5 *μ*l water, 5 *μ*l of 2.5 mM dNTPs, 5 *μ*l of 20 mM MgCl_2_ PCR buffer, 0.5 *μ*l of AmpliTaq Gold (Applied Biosystems, Darmstadt, Germany) and 1 *μ*l each of primers V*γ*1f, V*γ*10, J*γ*1.1/2.1 and FAM-labelled J*γ*1.3/2.3. For tube B, 10 *μ*l of DNA was mixed with the same reagents, but with different primers: 1 *μ*l each of V*γ*9, V*γ*11, J*γ*1.1/2.1 and FAM-labelled J*γ*1.3/2.3. The PCR reactions were run on a PTC200 thermal cycler (MJResearch/Biozym, Hess. Oldendorf, Germany) using the following protocol: denaturation at 94°C for 7 min, followed by 40 cycles of (1) 94°C for 1 min, (2) 60°C for 1 min and (3) 72°C for 1 min, and a final extension step of 72°C for 7 min. The PCR products were analysed together with a differentially labelled size standard on a Applied Biosystems 3100XL Genetic analyser (Applied Biosystems) and the resulting capillary electropherogram traces were evaluated for the occurrence of distinct multiple (rarified) or single (clonal) peaks in the range of 150–250 base pairs (tube A products) and 110–210 base pairs (tube B products) (van Dongen *et al*, 2003). For statistical analyses, cases were separated into those with polyclonal TCR V*γ* gene rearrangements, and those with rarified and/or clonal TCR V*γ* gene rearrangements.

### Statistical evaluation

Correlations between parameters were determined using the Pearson's correlation coefficient. The ANOVA, *t*-test or Mann–Whitney test were applied to compare all continuous parameters either for different classes or for two-class cases. Frequency tables were tested by the *χ*^2^-test for the comparison of discrete parameters. Stepwise Cox-regression analysis was used to test the significance of parameters with survival time or DFS time (Maximum Likelihood estimates, Wald and *χ*^2^-test). For discrete parameters, Kaplan–Meier curves for different strata were plotted for DFS and OS. The log-rank test was used to test the significance of two survival curves. The tests were two-sided and *P*-values <0.05 were considered to be statistically significant and are referred to as ‘correlation’, whereas *P*-values of 0.05–0.1 are considered as ‘trend’ and termed as ‘trend of a correlation’. All statistical evaluations were carried out using statistical program package SAS (SAS Institute, Inc. Cary; NC, USA).

## Results

### Characterisation of lymphocyte infiltration in ovarian cancers

**Figure 1:**
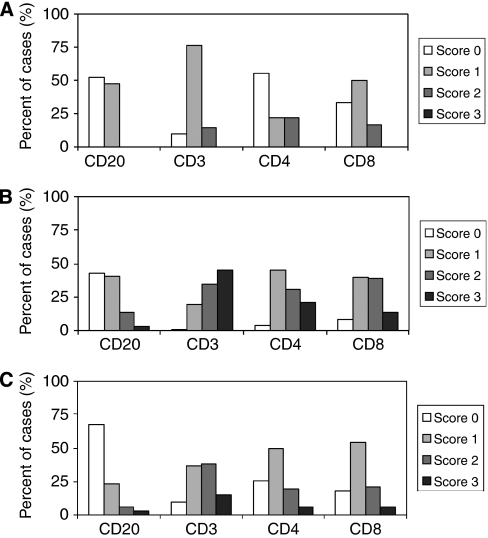
Summary of lymphocyte infiltration in ovarian carcinomas. The graphs provide the distribution of immunohistochemical scores, that is, lymphocyte infiltration (given as the percentage of cases with each IHC score on the y axis; Materials and Methods) in non-neoplastic ovaries (**A**), as well as for stromal (**B**) and intraepithelial lymphocytes infiltration (**C**) in ovarian carcinomas. Note the increased presence of CD3-positive as compared with CD4- and CD8-positive T lymphocytes in ovarian carcinomas (shift of bars to right; refer to text for *P*-values).

**Figure 2:**
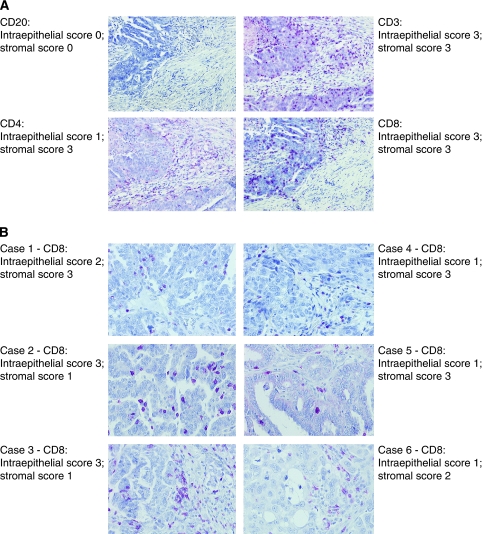
Representative immunohistochemical staining of lymphocytes in ovarian carcinomas. (**A**) Provides representative serial sections of an ovarian carcinoma stained for CD20, CD3, CD4 and CD8, with IHC scores given below the photographs (magnification × 100). (**B**) Shows representative IHC stainings of CD8-positive T lymphocytes in six cases, with high (case #1, 2, 3 (left)) and low (case #4, 5, 6 (right)) intraepithelial T-lymphocyte infiltration (magnification × 100), with the IHC score given beside the photographs. Note that two cases exhibited rarified/clonal (case #1) and polyclonal (case #4) T-cell receptor *γ* (TCR*γ*) rearrangements (refer to Figure 4A).

Immunohistochemical staining and evaluation were successful for CD20 in 97 out of 100 (97%) cases, for CD3 in 92 out of 100 (92%) cases, for CD4 in 86 out of 100 (86%) cases and for CD8 in 97 out of 100 (97%) cases. A complete or partial loss of tumour area after the staining procedure of TMAs was responsible for the failure of evaluation in some samples. Figure 1 provides a summary of the evaluated lymphocyte infiltration in non-neoplastic ovaries, and for stromal and intraepithelial tumour areas in ovarian carcinomas. Representative photographs of immunohistochemical stainings are given in Figure 2.

In non-neoplastic ovaries, CD20-, CD3-, CD4- and CD8-positive lymphocytes were present at low numbers in 47.8, 90.5, 44.4 and 66.7% of cases, respectively.

In ovarian carcinomas, stromal CD20-, CD3-, CD4- and CD8-positive lymphocytes were detected in 57.7, 99.0, 96.4 and 91.7% of cases and intraepithelial CD20-, CD3-, CD4- and CD8-positive lymphocytes were detected in 33.0, 90.2, 74.4 and 81.4% of cases, respectively. In contrast to non-neoplastic ovaries, ovarian carcinomas were predominantly infiltrated by higher numbers of lymphocytes. Statistical comparison of lymphocyte subsets between non-neoplastic ovaries and either stromal or intraepithelial ovarian carcinomas revealed significantly elevated CD3 (*P*<0.001), CD4 (*P*=0.002) and CD8 (*P*<0.001) positive stromal lymphocytes or significantly elevated CD20 (*P*=0.001), CD3 (*P*<0.001), CD4 (*P*<0.001) and CD8 (*P*<0.001) positive intraepithelial lymphocytes in ovarian carcinomas. There was no difference between the presence of CD20-positive lymphocytes in non-neoplastic ovaries and stromal tumour areas (*P*=0.136).

### Lymphocyte infiltration influences the survival of ovarian carcinoma patients

**Figure 3:**
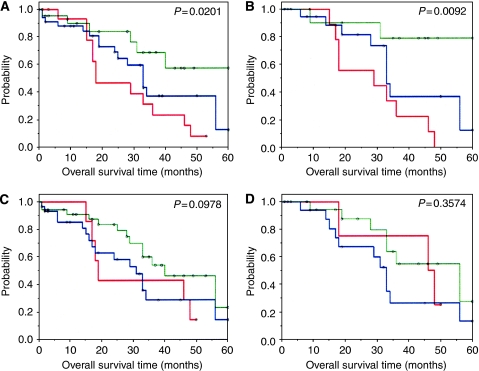
: Correlation of CD3- and CD8-positive T-lymphocyte infiltration of stage III ovarian carcinomas with clinical follow-up. The panels provide results of Kaplan–Meier analyses for overall survival with respect to intraepithelial (**A** and **B**) and stromal (**C** and **D**) CD8-positive T lymphocytes in stage III patients with optimal debulking (**A** and **C**), as well as for stage III patients with optimal debulking and adjuvant paclitaxel/carboplatin chemotherapy (**B** and **D**). Significant correlation to improved overall survival was only seen for increasing numbers of intraepithelial CD8-positive T lymphocytes (**A** and **B**; refer also to main text). Coding: red lines=IHC score 0 (no tumour-infiltrating lymphocytes (TILs)), blue lines=IHC score 1 (<5 TILs) and green lines=IHC scores 2+3 (>5 TILs).

To evaluate whether the presence of lymphocytes is of prognostic value, statistical analyses (Figure 3) were performed with respect to DFS and OS within all stage III patients (*n*=100), in stage III patients with optimal debulking (*n*=72), as well as in stage III patients with optimal debulking and undergoing adjuvant paclitaxel/carboplatin therapy (*n*=43).

Using the log-rank test to determine the significance of lymphocyte infiltration for survival, only stromal (*P*=0.0534) and intraepithelial (*P*=0.0402) CD3-positive T cells showed a trend and significant correlation with DFS for stage III patients with optimal debulking, respectively.

However, as this did not reflect the increasing numbers of T cells being linked to increasingly better survival, we performed an additional Cox-regression analysis. This analysis revealed a significant prognostic impact of increasing CD8-positive lymphocyte infiltration in long-term OS, that is, the effect only becoming apparent after a lag phase of about 1 year after surgery (Figure 3). Importantly, increasing numbers of intraepithelial CD8-positive T lymphocytes were significantly associated with increasingly improved OS 15 months after surgery for all optimally debulked stage III patients (*P*=0.0201), as well as for optimally debulked stage III patients who had received adjuvant paclitaxel/carboplatin chemotherapy (*P*=0.0092) (Figure 3A and B). In contrast, stromal CD8-positive T lymphocytes had no influence on OS in these patient sub-groups (all optimally debulked stage III patients: *P*=0.0978; optimally debulked stage III patients having received paclitaxel/carboplatin therapy: *P*=0.3574; Figure 3C and D).

Finally, multivariate stepwise Cox-regression analysis for OS, with all clinico-pathological and experimental parameters, validated debulking surgery (*P*<0.0001; hazard ratio (HR)=0.231; 95% confidence=0.116–0.462) and type of adjuvant chemotherapy (*P*=0.0014; HR=2.923; 95% confidence=1.513–5.648), as well as intraepithelial infiltration of CD8-positive T lymphocytes (*P*=0.0005; HR=0.408; 95% confidence=0.246–0.678) as significant prognostic factors. In this, optimal debulking surgery, adjuvant paclitaxel/carboplatin therapy and high numbers of intraepithelial CD8-positive T lymphocytes were significantly associated with improved survival.

### Analysis of TCR*γ* gene rearrangements

To assess whether TILs in ovarian carcinomas carried clonal TCR gene rearrangements, thereby indicating a potential recognition of a specific tumour cell antigen, we analysed TCR*γ* genes using the Biomed-2 protocol (van Dongen *et al*, 2003). Appropriate DNA and PCR quality was obtained from 93 out of 100 (93%) ovarian carcinomas and from 19 out of 24 (79%) non-neoplastic ovaries.

**Figure 4:**
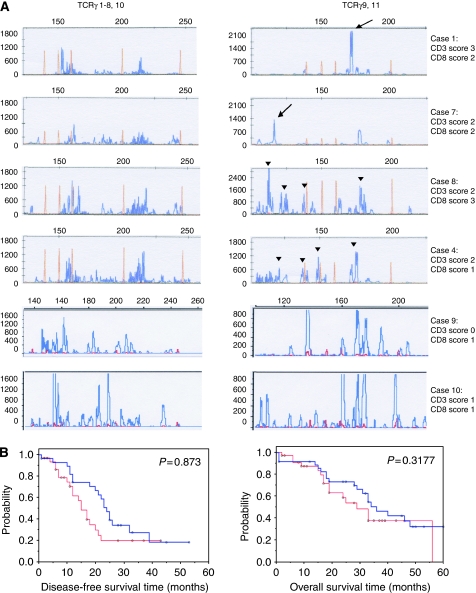
Analysis of T-cell receptor *γ* (TCR*γ*) gene rearrangements and association with survival. (**A**) Provides electropherogram traces of the PCR-based TCR*γ* gene rearrangement analysis (Materials and Methods) (van Dongen *et al*, 2003) for V*γ*1-8,10 (left) and V*γ*9,11 (right) in four ovarian carcinomas (cases 1, 4, 7 and 8; cases 1 and 4 are the same as in Figure 2B) and two non-neoplastic ovaries (cases 9 and 10). Cases 1 and 7 show polyclonal TCR*γ* gene rearrangements for V*γ*1-8,10 (left) and rarified/clonal TCRV*γ* gene rearrangements for V*γ*9,11 (right, black arrows). Cases 8 and 4 show polyclonal TCR*γ* gene rearrangements for V*γ*1-8,10 (left) and oligoclonal/rarified TCRV*γ* gene rearrangements for V*γ*9,11 (right, black arrowheads). The non-neoplastic ovaries (cases 9 and 10) show polyclonal/oligoclonal TCR*γ* gene rearrangements. The score for intraepithelial CD3- and CD8-positive T-cell infiltration of each case is given beside the corresponding graphs. In the eletropherogram traces, the top x axis indicates the size range of PCR products in base pairs and the y axis indicates the intensity of the PCR product. Blue lines/peaks represent labelled, TCR*γ*-specific PCR products and red lines/peaks represent the standard size marker. (**B**) Provides the results of Kaplan–Meier analyses for disease-free (left) and overall (right) survival of all cases with polyclonal (red line) or rarified/clonal (blue line) TCR*γ* gene rearrangements. Note the trend towards early improved disease-free survival for cases with rarified/clonal TCR*γ* gene rearrangements.

In non-neoplastic ovaries, we observed poly- and ‘oligoclonal’, that is, ‘rarified’ as compared with typical polyclonal, TCR*γ* gene rearrangements in 10 out of 19 (52.6%; V*γ*1-8,10) and 9 out of 19 (47.4%; V*γ*9,11) cases (Figure 4A). As only low numbers of T lymphocytes had been detected (Figure 1) in these non-neoplastic ovaries, the oligoclonal pattern might simply reflect ‘pseudoclonality’, that is, the detection of single or few T lymphocytes not related to an antigen-specific response.

In ovarian carcinomas of stage III patients (*n*=93), polyclonal TCR*γ* gene rearrangements were observed in 41 out of 93 (44.1%) cases, rarified TCR*γ* gene rearrangements in 37 out of 93 (39.8%) cases and clonal TCR*γ* gene rearrangements in 15 out of 93 (16.1%) cases (Figure 4A). Clonal TCR*γ* gene rearrangements were detected for V*γ*1-8 or V*γ*10 chains in 6 out of 93 (6.5%) cases and for V*γ*9 or V*γ*11 chains in 9 out of 93 (9.7%) cases. Finally, there was a trend of a correlation of rarified/clonal TCR*γ* gene rearrangements with improved DFS (*P*=0.0873), but not OS, (*P*=0.3177) in patients with stage III ovarian carcinomas and optimal debulking surgery (Figure 4B).

### Investigation of Her2/neu expression, TILs and TCR*γ* restriction in ovarian carcinomas

The Her2/neu protein expression has been associated with clonal T lymphocytes in breast cancer patients (Peoples *et al*, 1995; Goodell *et al*, 2008; Wiech *et al*, 2008), and strategies of immunotherapy eliciting antibody and CD8-positive T-cell responses against Her2/neu are being discussed (Disis *et al*, 2002). Therefore, we next investigated the Her2/neu protein expression in a subset of patients.

A positive Her2/neu protein expression was found in 37 out of 55 (67.2%) cases, with a Her2/neu score of 2 in 12 out of 55 (21.8%) and a score of 3 in 3 out of 55 (5.5%) cases. In this series of cases, the Her2/neu expression did not have any prognostic value in the different sub-groups of patients studied. Moreover, Her2/neu expression was linked to intraepithelial (*P*=0.0264), but not stromal (*P*=0.1418), CD8-positive T-lymphocyte infiltration. Intriguingly, this correlation was non-directional, and increasing Her2/neu scores were marginally associated with lower numbers of intraepithelial CD8-positive T-lymphocyte infiltration (*R*=−0.257; *P*=0.0626). There was also no significant correlation of HER2 expression with TCR*γ* restriction (*P*=0.7245).

## Discussion

In this study, we further clarified and validated the prognostic significance of TILs in ovarian carcinoma, by focussing on the exact location of TILs within the tumour mass, in a homogeneous group of serous stage III ovarian carcinoma patients and the clinically important sub-groups of optimally debulked and paclitaxel/carboplatin-treated patients. Furthermore, we provide data supporting the concept that the presence and persistence of lymphocytes within ovarian carcinomas may be triggered by clonal selection and/or restriction of T lymphocytes against specific (tumour cell) antigens. We conclude that such T lymphocytes infiltrating and persisting in the tumour parenchyma may be involved in an anti-tumoural response and may represent a basis for the further development of immune-mediated therapy.

In the past decade, several investigators have recognised the immune system and, in particular, the presence of TILs as a valuable, clinically relevant prognostic marker and as an immunological basis for the development of novel therapeutic strategies in patients with epithelial cancers (Parmiani, 2005) such as adenocarcinomas of the lung (Dieu-Nosjean *et al*, 2008), the colorectum (Galon *et al*, 2006) and the breast (Sheu *et al*, 2008).

In the case of ovarian carcinomas, TILs have been characterised and further specified in functional terms in a variety of studies, and the general consensus is that the presence of T lymphocytes has a major impact on the progression and clinical course of the disease. In particular, it was shown that high numbers of CD3-positive T cells are indicative for improved survival (Zhang *et al*, 2003; Curiel *et al*, 2004; Raspollini *et al*, 2005; Sato *et al*, 2005; Hamanishi *et al*, 2007; Tomsová *et al*, 2008; Clarke *et al*, 2009; Leffers *et al*, 2009) and that this effect is mediated preferentially by the CD8-positive (cytotoxic) subtype of CD3-positive T lymphocytes (Sato *et al*, 2005; Hamanishi *et al*, 2007; Clarke *et al*, 2009; Leffers *et al*, 2009). In contrast, the presence of the CD4-positive regulatory subtype of T lymphocytes (Treg; CD4+CD25+FoxP3+) seems to reduce tumour-specific immunity and results in poorer survival of patients with ovarian carcinomas (Curiel *et al*, 2004). Moreover, as observed for colorectal cancers (Galon *et al*, 2006), the location of TILs within the tumour mass of ovarian carcinomas has been shown to be important for revealing a prognostic effect (Zhang *et al*, 2003; Sato *et al*, 2005; Clarke *et al*, 2009).

Several issues, however, still remained unclear, especially whether the exact localisation of TILs does influence their prognostic/predictive value and whether the TILs detected within ovarian carcinomas are clonally restricted and respond to a specific tumour-specific antigen, such as the candidate protein Her2/neu (Peoples *et al*, 1995; Goodell *et al*, 2008; Wiech *et al*, 2008). In this study, we addressed these issues in a group of stage III ovarian carcinoma patients, further stratified into clinically relevant sub-groups of optimally debulked or optimally debulked and adjuvant paclitaxel/carboplatin-treated patients.

Similar to previous studies (Zhang *et al*, 2003; Curiel *et al*, 2004; Raspollini *et al*, 2005; Sato *et al*, 2005; Hamanishi *et al*, 2007; Tomsová *et al*, 2008; Clarke *et al*, 2009; Leffers *et al*, 2009), we evaluated the number and exact localisation of TILs within ovarian carcinomas and included the entire information in statistical analyses, without using a simplified cutoff for correlation of ‘negative’ or ‘positive’ TILs with patient survival. Particularly, we defined ‘stromal’ TILs as those located in tumour-associated stroma and/or in perivascular spaces, and ‘intraepithelial’ TILs as those clearly leaving the vascular/perivascular spaces to infiltrate into the tumour cell parenchyma. As we analysed representative tissue specimens from the central areas of only serous stage III ovarian carcinomas, selected by an experienced pathologist to reflect about 60% tumour and 40% stroma content, the distribution of tumour/stroma was taken into account. Similar to the study by Zhang *et al* (2003), we correlated the presence of different numbers of stromal and intraepithelial CD20-, CD3-, CD8- and CD4-positive TILs to clinico-pathological variables and survival in a homogeneous group of patients. With this approach, we could further validate that the presence of high numbers of intraepithelial, but not stromal, CD3-positive T lymphocytes was associated with an improved DFS when examining all stage III patients with optimal debulking surgery. Furthermore, we did not find a significant correlation between the number of CD4-positive T lymphocytes and poor survival, as observed by the specific analysis of the CD4+CD25+FOXP3+ Treg subset of T lymphocytes (Curiel *et al*, 2004). The analysis of all CD4-positive T lymphocytes in our study may, however, have hidden such a correlation, especially as CD4 staining alone rather detects the large majority of CD4-positive helper T cells than the minority of Tregs. More importantly, we confirmed and extended previous studies on the role of CD8-positive TILs (Sato *et al*, 2005; Hamanishi *et al*, 2007; Clarke *et al*, 2009; Leffers *et al*, 2009). The presence of high numbers of intraepithelial, but not stromal, CD8-positive T lymphocytes was of uni- and multivariate prognostic benefit for stage III ovarian cancer patients, especially in the large sub-group of patients receiving adjuvant paclitaxel/carboplatin therapy. Interestingly, Tsuda *et al*, 2007 showed that Taxol stimulates the immunogenic potential of CD8-positive T cells *in vitro*, an effect that may be further exploited in current therapeutic studies (Disis *et al*, 2002).

In continuation, we tested the hypothesis that the intraepithelial presence and persistence of T lymphocytes is mediated by the recognition of (a specific) tumour-cell-specific antigen and clonal expansion of (CD8 positive) TILs. Indeed, we showed that lymphocytes infiltrating stage III ovarian carcinomas are shifted to rarified or clonal TCR*γ* gene rearrangements, an indication of the expansion of individual T-cell clones, as can be also observed for T-cell malignancies (van Dongen *et al*, 2003). Clonal restriction occurred for TCR V*γ*1-8 or V*γ*10 chains in 6 out of 93 (6.5%) and for V*γ*9 or V*γ*11 chains in 9 out of 93 (9.7%) cases. Our data extend and specify previous findings by Raspollini *et al* (2005), who described positive PCR products for TCR V*γ* gene rearrangements in 31.3% of serous ovarian carcinomas, without stratifying poly-, oligo- or monoclonal TCR*γ* gene rearrangements. Thus, the latter study showed that the mere presence of TCR*γ* gene rearrangements, that is, the detection of PCR products, correlated to DFS, which was explained to be because of the presence of T cells expressing TCR*γδ* proteins. However, as TCR *β*, *δ*, *γ* gene rearrangements occur in all T cells, but are not all productive for protein expression, DNA-based TCR*γ* PCR analysis merely provides information regarding the clonality of lymphocytes. Our study now provides further insights into this issue and shows that the presence of T cells with rarified and/or clonal TCR*γ* gene rearrangements may have a prognostic benefit, suggesting a targeted immune response against ovarian carcinomas.

Nevertheless, as restricted usage of TCR V*γ*-J*γ* gene rearrangements may also occur in normal individuals (Kohsaka *et al*, 1993) and as TCR*β* gene rearrangements (Peoples *et al*, 1993), which may even further support the readout of clonality analysis (van Dongen *et al*, 2003), were not studied here, the results should be interpreted carefully. In fact, the observed ‘clonal’ restriction of TCR*γ* gene rearrangements may either point towards infiltration of ovarian carcinomas by one or few *αβ* T cells with restricted TCR*γ* gene rearrangements, or by one or few *γδ* T cells. In our study, the infiltration of CD8-positive lymphocytes was of prognostic benefit. As CD8 expression is not observed in classical *γδ* T cells, our results suggest that the presence of clonal CD8-positive *αβ* T cells may be primarily involved in a possible anti-tumoural immune response in ovarian carcinomas. Our data encourage further advanced studies on the nature of TIL clonality in ovarian carcinomas, for example, also including complementary determining region (CDR3) spectratyping of (single) microdissected TILs and reconstitution of TCR chains (Hofbauer *et al*, 2003; Seitz *et al*, 2006).

In view of the potential clonal restriction of CD8-positive T lymphocytes actively counteracting the tumour and its progression, we investigated whether the tumour-cell-specific expression of Her2/neu protein in ovarian carcinomas may represent a candidate target antigen of TILs, as suggested for breast cancer (Peoples *et al*, 1995; Goodell *et al*, 2008; Wiech *et al*, 2008) and as shown in a murine model of ovarian cancer (Yang *et al*, 2007). However, the correlation of Her2/neu protein expression to the presence of TILs and TCR*γ* gene rearrangements in our group of ovarian carcinomas revealed that this is not the case, suggesting that other tumour antigens may be more promising candidate target antigens. Indeed, Milne *et al* (2008) have proposed NY-ESO-1, a 22 kDa protein encoded on chromosome Xq28 and belonging to the so-called ‘cancer-testis antigen’, as the target antigen of TILs in ovarian carcinomas. Clearly, further detailed studies are needed to clarify the role of CD8-positive T cells in ovarian carcinoma and their role in a possible anti-tumoural response *in situ*.

In summary, our study provides evidence that intraepithelial infiltration of ovarian carcinomas by CD8-positive T lymphocytes is prognostic for improved survival in optimally debulked, stage III ovarian cancer patients, most significantly also for those with the option of adjuvant paclitaxel/carboplatin therapy. Persistence of these T lymphocytes within the tumour parenchyma, potentially reflecting a T lymphocyte-triggered anti-tumoural immune response, is essential for the beneficial prognostic effect. This may be further exploited for supportive and/or novel immunotherapy.

## Figures and Tables

**Table 1:** Clinico-pathological parameters of investigated ovarian carcinomas

	***n***	**%**
Patient age (years)	64 (32–85)	
		
*FIGO stage*		
IIIB	4	4
IIIC	96	96
		
*Tumour histology*		
Serous	100	100
		
*Tumour grading*		
1	1	1
2	31	31
3	66	66
4	2	2
		
*Debulking status*		
>1 cm	25	26
<1 cm	72	74
		
*Adjuvant therapy*		
None	7	7
Paclitaxel/carboplatin	60	62
Other	30	31
		
*Clinical follow-up*		
Incidence of recurrence	52/77 (68%)	
Disease-free survival (median months)	15 (range 1–53)	
Incidence of death	55/97 (57%)	
Overall survival (median months)	19 (range 1–82)	
		

All patients were Caucasians and had not received any immunotherapy and/or HER2-targeted therapy. The term ‘disease-free survival’ and ‘overall survival’ denote the time from the primary resection of the ovarian carcinoma until the events of tumour recurrence and death due to ovarian cancer, respectively.
